# Annotations

**Published:** 1898-09-17

**Authors:** 


### Annotations.

### The Marriage of First Cousins.

A letter in the *Times* on the subject of Cousin Marriage in Fiji raises a question which ought to be capable of solution by a little inquiry in a country in which cousin marriages are so common as they are in England. It has often been asserted that the marriage of cousins is bad, an assertion which has just as often been met by actual examples of the fertility of such marriages, and of the health and vigour of their offspring. Yet instances in which the result has been disastrous continue to occur with just sufficient frequency to keep alive the tradition. Looking at the matter from the medical side, and arguing, it is to be feared, from analogy rather than from any great number of concrete examples, medical men have been in the habit of explaining such evil consequences, when they occur, on the hypothesis of their being caused by an accentuation of family taint by inbreeding. It is suggested, however, by Basil Thomson that it is not the cousinship which is the source of the evil, but the sex relationship of the parents of the cousins in question. Among certain tribes in Fiji a custom has for long existed which compels a man to choose his wife from among the daughters of his mother's brother. Thus in any family the male offspring of the female is mated with the female offspring of the male, and as the result of a census of a dozen villages it was found that these marriages were fertile, and that a large proportion of the children born to them were reared. In marriages of first cousins who were the offspring of two brothers or of two sisters, a relation which is looked upon in these tribes as incestuous, none of the children survived infancy. Is it possible, then, that the Fijians have stumbled upon a truth in regard to cousin marriages? It is an interesting question, and one to which, we fear, no positive answer can yet be given. It seems clear, however, that the peculiarities of a family are not transmitted equally by the male and by the female; and the distribution of such a disease as pseudo-hypertrophic paralysis—a disease of boys which is almost exclusively transmitted from the mother's side—is of itself sufficient to prevent us denying that the results of cousin marriages may, perhaps, be influenced by the sex relationship of the parents of the cousins in question.

### The Expert Witness.

A recent decision given in the High Court of the State of Illinois in regard to the duties of expert witnesses has drawn considerable attention to the question of expert evidence. According to the decision referred to, it is the duty of every citizen to answer to a subpœna, however little he may know about the case in question, and to apply such expert knowledge as he may possess to the solution of any hypothetical questions put to him in the witness-box, without any extra payment for his evidence. We do not wonder at the indignation expressed at such a condition of the law. When, however, medical men, in defending the rights and privileges of the expert medical witness, go so far as to claim that he may consistently and with propriety take a side, as has recently been done in an American contemporary, we must raise a protest. The witness in the box, whatever his position, whether he be lay or medical, whether he be an ordinary witness to fact or an expert witness to matter of opinion, has but one duty, namely, to speak the truth. It often happens, no doubt, that the lawyer in charge of a case requires the aid of a medical man in its preparation, and that even when in court a barrister may find it desirable to have at his elbow a skilled expert to whom he can appeal for information in regard to points as they arise. But a broad distinction must be drawn between the position of an expert so employed—employed much as the barrister might employ a book or any other source of knowledge—and that of an expert placed in the box to give evidence by which the jury is to be guided in its verdict. The barrister and his advising expert may be regarded as one—in fact, they are sometimes rolled up into one in the form of that hybrid being, a medical barrister; but we consider it most undesirable, although it is a thing that unfortunately is often done, that the same medical man should act both as witness and adviser. In any case, however, whatever the relation between the expert and the barrister or his client, the duty of the expert so soon as he enters the box is to speak "the truth, the whole truth, and nothing but the truth," and we protest against every suggestion that, when occupying that position, it is allowable for him to be in the slightest degree a partisan.

### Anarchism and Madness.

The whole civilised world has this week been shocked by the dastardly assassination of the Empress of Austria, as cold-blooded a murder as it is possible to conceive; and signs are not wanting that the civilised world may at last wake up to the fact that a cult which is responsible for such a series of crimes as can be laid to the charge of Anarchism is a distinct danger to civilisation itself. Yet already we hear the question asked, Was he not mad? and already we see it suggested that the aimlessness, the utter uselessness of the crime, and the callousness and outrageous levity of the criminal are to be taken as signs of his insanity. Perhaps, indeed, he was mad. He certainly did not regard his environment with normal eyes, and, even according to the legal criteria of "responsibility," it might be maintained that at the time of committing his crime he was not conscious of right or wrong—as, indeed, what Anarchist is? But is he therefore to go scot free? We have again and again expressed the opinion that the plea of insanity is no sufficient palliation for crime, and that the definition of responsibility has been somewhat strained of late; and while we are quite prepared to admit that Anarchism is a form of madness, and that Anarchists, who, by the bye, form a fairly numerous body, are practically insane so far as social questions are concerned, we, at the same time, maintain that this is a form of madness which can only be properly dealt with by the firm—the very firm—hand of the law, and that Anarchists are criminals in intent even before they break the law. It is not improbable that this miserable case may lead people to take a juster view of "responsibility" than has been fashionable in late years. The world has no room for a sect of lunatics whose madness takes the form of an attack upon society, and whose saints are those who by dagger and by dynamite take the lives of the noblest and the best.

